# T cell and cytokine signatures as early predictors of response to IL-12/IL-23 inhibition in Crohn’s disease

**DOI:** 10.3389/fimmu.2026.1753914

**Published:** 2026-03-04

**Authors:** Eduardo Martín Arranz, Laura García-Ramirez, Ana Van Den Rym, Cristina Suarez, Blanca García-Solís, Nestor Díaz-Iglesias, Alberto López-Lera, Fernando Corvillo, José Luis Rueda García, Paula Blanco-San Miguel, María Sánchez Azofra, Joaquín Poza Cordón, Yuval Itan, Eduardo López-Collazo, Rebeca Pérez de Diego, María Dolores Martín-Arranz

**Affiliations:** 1 Inflammatory Bowel Disease Unit, Gastroenterology Department, La Paz University Hospital, Madrid, Spain; 2 Group of Gastrointestinal Immune-Mediated Diseases IdiPAZ Institute for Health Research, La Paz University Hospital, Madrid, Spain; 3 Laboratory of Immunogenetics of Human Diseases, IdiPAZ Institute for Health Research, La Paz University Hospital, Madrid, Spain; 4 Innate Immunity Group, IdiPAZ Institute for Health Research, La Paz University Hospital, Madrid, Spain; 5 Interdepartmental Group of Immunodeficiencies, Madrid, Spain; 6 IdiPAZ Institute for Health Research, La Paz University Hospital, CIBERER U-754, Madrid, Spain; 7 The Charles Bronfman Institute for Personalized Medicine and Department of Genetics and Genomic Sciences, Icahn School of Medicine at Mount Sinai, New York, NY, United States; 8 CIBER de Enfermedades Respiratorias (CIBERES), Instituto de Salud Carlos III, Madrid, Spain; 9 School of Medicine, Universidad Autónoma de Madrid, Madrid, Spain

**Keywords:** Crohn’s disease, flow cytometry, IL12/IL23 inhibitor, inflammatory bowel disease, T cells

## Abstract

**Introduction:**

Crohn’s disease (CD) is a disabling inflammatory disorder with highly variable response to biologic therapies. Despite the availability of multiple biologic agents, a significant proportion of patients experience primary non-response, while others initially respond but subsequently develop secondary loss of response, leading to sequential treatment failures and increased healthcare burden. The disease course and response to treatment are remarkably heterogeneous among patients, and a better understanding of the personalised pathways affected is required. Identifying early biomarkers of treatment efficacy is crucial for improving patient outcomes and reducing unnecessary healthcare costs.

**Methods:**

In this study, we comprehensively analysed T cell subpopulations in blood and lamina propria, together with serum cytokine profiles, in CD patients receiving the IL-12/IL-23 inhibitor ustekinumab.

**Results:**

The most remarkable findings were observed in peripheral blood. At week 24, good responders showed decreased Th1, increased Th2, and reduced IL-17A serum levels compared with non-responders. Importantly, elevated IL-12/IL-23 serum levels at week 8 associated with favourable clinical and endoscopic outcomes, suggesting effective pathway blockade.

**Discussion:**

These findings support the measurement of serum IL-12/IL-23 at week 8 as a simple, early predictor of ustekinumab response in CD, potentially guiding personalised treatment strategies and ultimately long-term response.

## Introduction

Inflammatory bowel disease (IBD) refers to a group of chronic idiopathic conditions affecting the gastrointestinal tract (GI), sometimes involving other organs. Hence, they are considered systemic diseases. They are characterised by severe inflammation of the small intestine and/or colon which, among other symptoms, produces recurrent diarrhoea and abdominal pain (1, 2). Within IBD, two clinical entities have been classified: Crohn’s disease (CD) and ulcerative colitis (UC). CD is a recurrent inflammatory disease that can affect any point of the gastrointestinal tract, from the mouth to the anus, and whose cardinal symptoms are diarrhoea, abdominal pain and weight loss, although others such as fever, rectal bleeding and intestinal obstruction can also occur. It affects the wall of the digestive tract transmurally, which can lead to fistulas, abscesses and strictures. It also has extraintestinal cutaneous manifestations such as pyoderma gangrenosum and erythema nodosum, articular manifestations such as arthralgias and arthritis and ocular manifestations such as uveitis, among others. Its incidence and prevalence are increasing in all ethnic groups. The pathogenesis of IBD is only partially understood, but several environmental and host factors are involved. Intestinal inflammation arises due to abnormal host-microbiota interaction. Disturbances to homeostatic coexistence are influenced by host genetic factors, proper functioning of the epithelial mucosal barrier, innate and adaptive immunity, and qualitative and quantitative changes in the composition of the microbiota (1, 3–5). Genome-wide association (GWA) studies have provided a broad view of the genomic contribution of several loci. The genetic contribution to CD is estimated at 50%. In general, the main genetic association can be divided into genes contributing to the innate response and those contributing to the acquired immune response. Certain genes have been implicated in CD, such as interleukin (IL)-23 pathway genes (IL23R, IL12B, STAT and the transcription factor NKX2-3) (6). Recent studies have focussed on environmental modulation that generates epigenetic changes as contributors to the pathology. Relevant epigenetic mechanisms have been found in IBD biology, including immune dysregulation, pathogen-host recognition and mucosal integrity (7–10).

One of the most relevant immune cells linked to the pathogenesis of IBD are T cells. T-helper (Th) and T-regulatory (Treg) cells are the main actors involved. Although the pathogenesis of IBD is not yet clear, studies have shown that the imbalance between Th17 and Treg cells contributes to IBD (11). In addition, a dual role of Th17 cells has been described in the pathogenesis of IBD (12). CD4+ Th17 cells can not only protect the intestinal mucosa by maintaining the balance of the immune microenvironment but also exacerbate the intestinal inflammatory response through proinflammatory cytokines. In addition, Treg cells are a subset of CD4+ T cells, which play a negative immunomodulatory role and are essential in maintaining immune tolerance and balance. Tregs cells mainly participate in various immune diseases by secreting anti-inflammatory cytokines, such as TGF-β and IL-10, suppressing the activity of immune cells and thereby controlling inflammation (11). Th17 and Treg cells are related through differentiation and function inhibition. They share a common signal pathway mediated by TGF-β. Studies have shown that in the presence of IL-6 or IL-21 (with TFG-β), naïve CD4+ T cells differentiate into Th17 cells; however, in the absence of pro-inflammatory cytokines, naïve CD4+ T cells differentiate into Treg cells (13). Once this balance is broken, a number of autoimmune diseases occur, including CD (11). More than 20 years ago it was known that multiple components of the IL-12/IL-23 signalling pathway are involved in CD (14). IL-12 (derived from dendritic cells (DC) and macrophages) provides an important regulatory link between innate and adaptive immunity. It performs several biological functions, including the differentiation of naïve CD4+ T cells into IFN-γ-producing Th1 cells (15).

Treatment of IBD has been revolutionised by the introduction of therapeutic antibodies: anti-tumour necrosis factor (TNF)-α, anti-α4β7, anti-IL-23/IL-12 and more recently small molecules as JAK inhibitors (16–20). Disease course and treatment response are remarkably heterogeneous among individual patients and better understanding of the personalised pathways affected is required, together with the discovery of biomarkers with high a prognostic value for a personalised treatment response (21–23). To this end, we decided to carry out a comprehensive cytometric study to analyse T cell subpopulations in peripheral blood and the lamina propria, as well as cytokine patterns during the course of treatment with anti-IL-23/IL-12 antibodies in CD, in order to identify biomarkers to predict the response to the treatment.

## Materials and methods

### Study approval

The experimental protocol was approved by the Ethics Committee of La Paz University Hospital (Madrid, Spain) (local code PI-4732) and written informed consent was obtained from the patients and healthy donors for participation in this study.

### Patient selection

From the induction cohort, a longitudinal cohort of CD patients with inflammatory activity was prospectively selected, who initiated treatment with Ustekinumab for remission induction, as chosen by their physician. Patients received their first dose of ustekinumab intravenously, adjusted by weight (6mg/kg: < 55 kg, 260 mg; 55–85 kg, 390 mg; > 85 kg, 520 mg) and subsequently followed maintenance treatment with subcutaneous ustekinumab 90 mg every 8 to 12 weeks at the discretion of their physician.

### Clinical, biochemical and endoscopic assessments

Clinical activity was assessed using the Harvey-Bradshaw Index (HBI). Clinical activity was defined as a decrease of ≥ 3 points from baseline in the HBI score, and clinical remission as a HBI score <5. Quality of life was also assessed using the Inflammatory Bowel Disease Questionaire-9 (IBDQ-9).

Biological activity was considered as increased faecal calprotectin (≥ 250µg/g; ≥ 100 in the case of interventional CD). Biological remission was defined as faecal calprotectin level < 250µg/g (< 100µg/g in patients with ileocecal resection). In patients with a baseline faecal calprotectin level < 250µg/g, biological remission was defined as a C-reactive protein value < 5g/dL.

Endoscopic activity was assessed using the Simple Endoscopic Score for Crohn´s Disease (SES-CD). Endoscopic response was defined as a ≥ 50% reduction in the SES-CD score from baseline, and endoscopic remission as a score ≤ 2. In patients with ileocelcal resection, postoperative recurrence was assessed using the Rutgeerts index. Postoperative recurrence was defined as a Rutgeerts score ≥ i2, endoscopic response as a decreased of ≥ 1 point, and endoscopic remission as a score ≤ i1.

At week 24, patients were classified into four response groups: Group 1, patients achieving clinical, biochemical and endoscopic remission; Group 2, patients with combined clinical and biochemical remission but persisting endoscopic activity; Group 3, patients achieving only one domain of remission (either clinical or biochemical) without endoscopic remission; and Group 4, patients without clinical, biochemical or endoscopic remission.

### Samples (serum samples and cell purification)

Serum samples were obtained from whole blood by centrifugation.

Peripheral blood mononuclear cells (PBMCs) were isolated using the Ficoll-Hypaque density gradient centrifugation (Amersham-Pharmacia-Biotech, Buckinghamshire, UK) of whole-blood samples obtained from the patients or healthy volunteers.

Colonoscopy sample processing for isolation of epithelial cells and immune cells from the lamina propria: biopsy samples were placed in Hank’s balanced salt solution (HBSS) without Ca^2+^ and Mg^2+^ (HBSS; Gibco, Grand Island, NY, USA) supplemented with 10% FCS (Gibco) and antibiotic/antimycotic (Gibco) for 30 min at 4 °C and gently stirred. The biopsy samples were then placed in HBSS without Ca^2+^ and Mg^2+^ 0.5mM EDTA (Gibco). The epithelial and immune cells were separated from the rest of the tissue by vigorous shaking at 37 °C in HBSS without Ca^2+^ and Mg^2+^0.5mM EDTA (Gibco). The biopsy tissues in HBSS were then cut with collagenase type IV (Gibco) and incubated for 30 min at 37 °C with shaking. After centrifuge the supernatant enriched with epithelial cells is discarded, leaving only the pellet of lamina propria cells.

### Flow cytometry study of lymphocyte subpopulations

The T-cell subpopulation samples were incubated with antibodies against LIVE/DEAD™ Fixable Yellow (Thermo Fisher Scientific, Waltham, *MA, USA*), CD3 FITC or BV510, CD4 BUV395 or APC, CD8 PerCP-Cy5.5, CD45RA BV650 or FITC, CD45RO BV510 or APC-Vio700, CXCR3 BV421, CCR6 BV605, CD25 BV786, CD127 PE and CD31 PE-Cy7 (BD Biosciences, San Jose, CA, USA) for 20 minutes. The cells were washed and resuspended in 0.2 mL 1x PBS for acquisition in a BD FACSCelesta™ Cell Analyser. The data were analysed with FlowJo™ v10.8.

Events were then gated on a lymphocyte gate based on forward-scatter (FSC-A) versus side-scatter area (SSC-A). Initial gating involved the use of a forward-scatter width (FSC-W) versus height (FSC-H) plot and side-scatter width (SSC-W) versus height (SSC-H) plot to remove doublets. Events were further gated on CD3, CD4 and CD8 for T cells. Following their identification, the T cells were analysed and classified into regulatory T cells, Th1, Th2 and Th17 cells, based on their expression of CD45RO, CD45RA, CD25, CD127, CXCR3 and CCR6. Flow cytometry gating strategy is described in **Supplementary Figure 1**.

### Cytokine determination

Cytokine production was assessed in serum samples in accordance with the manufacturer’s instructions and the concentration of IL-6, IL-17A and IL-12/IL-23 was determined by enzyme-linked immunoassay (ELISA) (Biolegend, San Diego, CA, USA).

### Statistical analysis

Mean values ± SD were calculated from the measurements of all patients in each group, which in turn were obtained from the mean of three independent experiments.

The Kruskal-Wallis test was used to analyse Th cells subpopulations, comparing the differences between healthy donors and Group 1 or 4 at 24 weeks of treatment.

For the comparison between weeks, Friedman’s test for dependent samples was used for studies of the IL-17-A cytokine, comparing the differences between week 0 and week 24 of treatment for each group. A mixed-effects model was used to analyse differences between groups (healthy controls and patient groups) over time (0, 8 and 24 weeks), with repeated measures taken from the same subjects. The Geisser-Greenhouse correction was applied and *post hoc* group comparisons at each time point were performed using Tukey’s multiple comparisons test for the studies of IL-12/IL-23 cytokines.

Differences between the samples were considered statistically significant if *p* < 0.05. The statistical analysis was performed with GraphPad Prism version 10.3.1.

## Results

### Patient selection and study strategy

23 patients selected for this study suffered from CD and had been treated with ustekinumab at the dosage indicated in Methods (see the Methods section) to study T cell subpopulations in PBMCs and the lamina propria at the start of treatment (0w), at 8 weeks (8w) and 24 weeks (24w). Cytokines levels (IL-6, IL-17A and IL-12/IL-23) were also measured at the same time. **Table 1A** shows the clinical data of the selected patients, **Supplementary Table 1** shows the analytical data and Four groups of patients were established (**Table 1B**) based on different criteria or response to treatment: Group 1 were patients with the best response to treatment, showing clinical, biological and endoscopic remission (**Tables 1B**, **Supplementary Table S2**); Groups 2 and 3 had an intermediate response to treatment (**Tables 1B**, **Supplementary Table S2**), showing both clinical and biological, but not endoscopic, remission (Group 2), or only one remission, clinical or biological, (Group 3); Group 4 had the poorest response to treatment, with no kind of remission at 24 weeks of treatment (**Tables 1B**, **Supplementary Table S2**). The baseline characteristics are summarised in **Supplementary Table 3**. **Supplementary Table 1** shows the analytical data of all the patients included in the study. It is worth mentioning that all the patients in Group 4 had been previously treated with adalimumab and were considered non-responders.

**Table 1:** Selection of patients and groups.

A		-30 days	Week 0	Week 8	Week 16		Week 24	Week 54
Informed consent		x						
Eligibility		x						
Weight			x	x	x		x	x
Size			x					
Concomitant medication			x	x	x		x	x
Adverse events			x	x	x		x	x
Colonoscopy		x					x	
Intestinal ultrasound		x		x	x		x	x
Conventional blood test			x	x	x		x	x
Faecal calprotectin			x	x	x		x	x
Levels of ustekinumab – Ac			x	x	x		x	x
HBI			x	x	x		x	x
IBDQ-9			x	x	x		x	x
Ustekinumab administration (induction)			x					
Ustekinumab administration (maintenance)				x	x		x	x
Lymphocyte subpopulations analysis in PBMC			x	x			x	
Lymphocyte subpopulations analysis in the lamina propria			x				x	
Measure of cytokines in serum			x	x			x	
B								
Name of group	Requirements of the group					Number of patients (n)		
Controls	Healthy donors							
1	Clinical, biological and endoscopic remission					2		
2	Clinical and biological remission (but not endoscopic remission)					5		
3	Clinical or biological remission					10		
4	No remission (neither clinical, nor biological, nor endoscopic)					6		

**(A)** Patient data collected at six time points. The lymphocyte subpopulation analysis in PBMC was performed at weeks 0, 8 and 24 of the study, and the lymphocyte subpopulation analysis in the lamina propria was performed at weeks 0 and 24. **(B)** The selection criteria of the groups analysed was based on remission (clinical, biological, or endoscopic). HBI, Harvey-Bradshaw Index. IBDQ-9, Inflammatory Bowel Disease Questionnaire-9. Green is group 1, yellow is group 2, orange is group 3 and red is group 4.

### Analysis of T cell subpopulations in the PBMC of CD patients treated with ustekinumab

**Figure 1:**
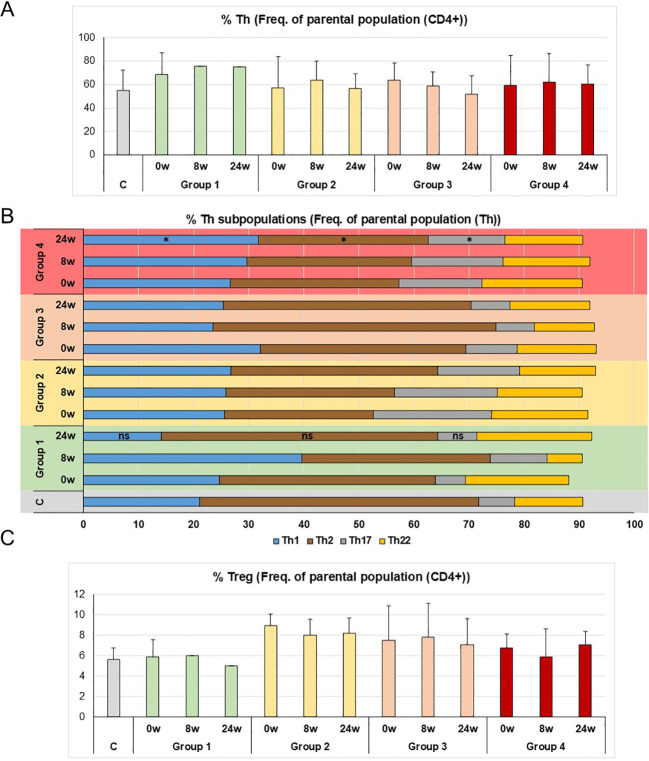
Distribution of T cells subpopulations in the PBMC of CD patients treated with ustekinumab. **(A)** Th cells as a percentage of the parental population, CD4+ cells. **(B)** Th1, Th2, Th17 and Th22 cells as a percentage of the parental population, Th cells. **(C)** Percentage of Treg cells as a percentage of the parental population, CD4+ cells. C is healthy donors, 0w, 8w and 24w are 0, 8 and 24 weeks of treatment, respectively. The mean ± SD values were calculated from the measurements of all patients in each group, which in turn were obtained from the mean of three independent experiments. The Kruskal-Wallis test was used for the studies of Th subpopulations, comparing the differences between the healthy controls and the patient groups at 24 weeks of treatment; *p* ≥ 0.9 (ns) for the differences in Th1, Th2 and Th17 between Group 1 and C; *p* = 0.05, *p* = 0.04 and *p* = 0.02 (*) for the differences in Th1, Th2 and Th17 between Group 4 and C respectively.

In order to obtain homogeneous data, CD patients with lymphopenia were excluded from the study (**Table 2**). We decided to study CD4+ T cell subpopulations in PBMC over a 24-week treatment period (0w, 8w and 24w). For the Th subpopulations, we analysed Th1, Th2, Th17 and Th22 cells (**Figures 1A, B**). As in previous studies (24), Th1 levels rose in the patients in Group 1 at 8 weeks of treatment, but levels dropped by 24 weeks. In general, all the groups had more Th1 cells than the controls, except Group 1 at 24 weeks of treatment, whose percentage of Th1 cells dropped, in a pattern similar to the controls, with no significant differences between the controls and Group 1 (**Figure 1B**). Unlike Group 1, Th1 levels increased in Group 4 over the weeks of treatment, showing opposite patterns in good and poor responders. These differences in Th1 levels were also found when compared with the controls (**Figure 1B**).

**Table 2:** Distribution of T-cell subpopulations in peripheral blood.

Description of the four groups of patients	Number of patients (n) analised	% CD4+ (Freq. of parental population (CD3+)) (range)
Healthy controls (n=7)		56.8-77.9
Group 1_Remission at three levels (clinical, biological and endoscopic) at 24 w of treatment	0 weeks (n=2)	41.5-47.5
	8 weeks (n=1)	48.3
	24 weeks (n=1)	23.2
Group 2_Remission at two levels (clinical and biological or clinical endoscopic) at 24 w of treatment	0 weeks (n=2)	48.9-77.3
	8 weeks (n=4)	44.3-77.1
	24 weeks (n=4)	54.7-60.6
Group 3_ Remission at one level (clinical or biological) at 24 w of treatment	0 weeks (n=7)	45.4-79.3
	8 weeks (n=8)	44.9-79.0
	24 weeks (n=8)	50.5-82.3
Group 4_ No remission at any level	0 weeks (n=3)	50.1-73.9
	8 weeks (n=6)	46.3-79.2
	24 weeks (n=5)	31.4-69.6

The values are presented as ranges of percentages of the parental population. Green is group 1, yellow is group 2, orange is group 3 and red is group 4.

With Th2 cells, levels were generally did not rise, unlike Th1 and Th17 cells in CD (25). We observed higher Th2 levels in the controls than in all the patient groups and, as expected, at 24 weeks of treatment, Group 1 (good responders) had recovered Th2 levels similar to the controls (**Figure 1B**), with significant differences compared to the controls (**Figure 1B**). This was not the case with Group 4, who showed significant differences in Th2 levels at 24 weeks of treatment compared to the controls (**Figure 1B**).

The study of Th17 cells showed that Group 1 had lower levels of these cells at 24 weeks of treatment, with similar percentages to the controls, there being no significant differences (**Figure 1B**). However, over the course of treatment, levels of Th17 did not fall in Group 4 patients, highlighting significant differences with the controls and indicating a poor response to treatment (**Figure 1B**).

**Figure 2:**
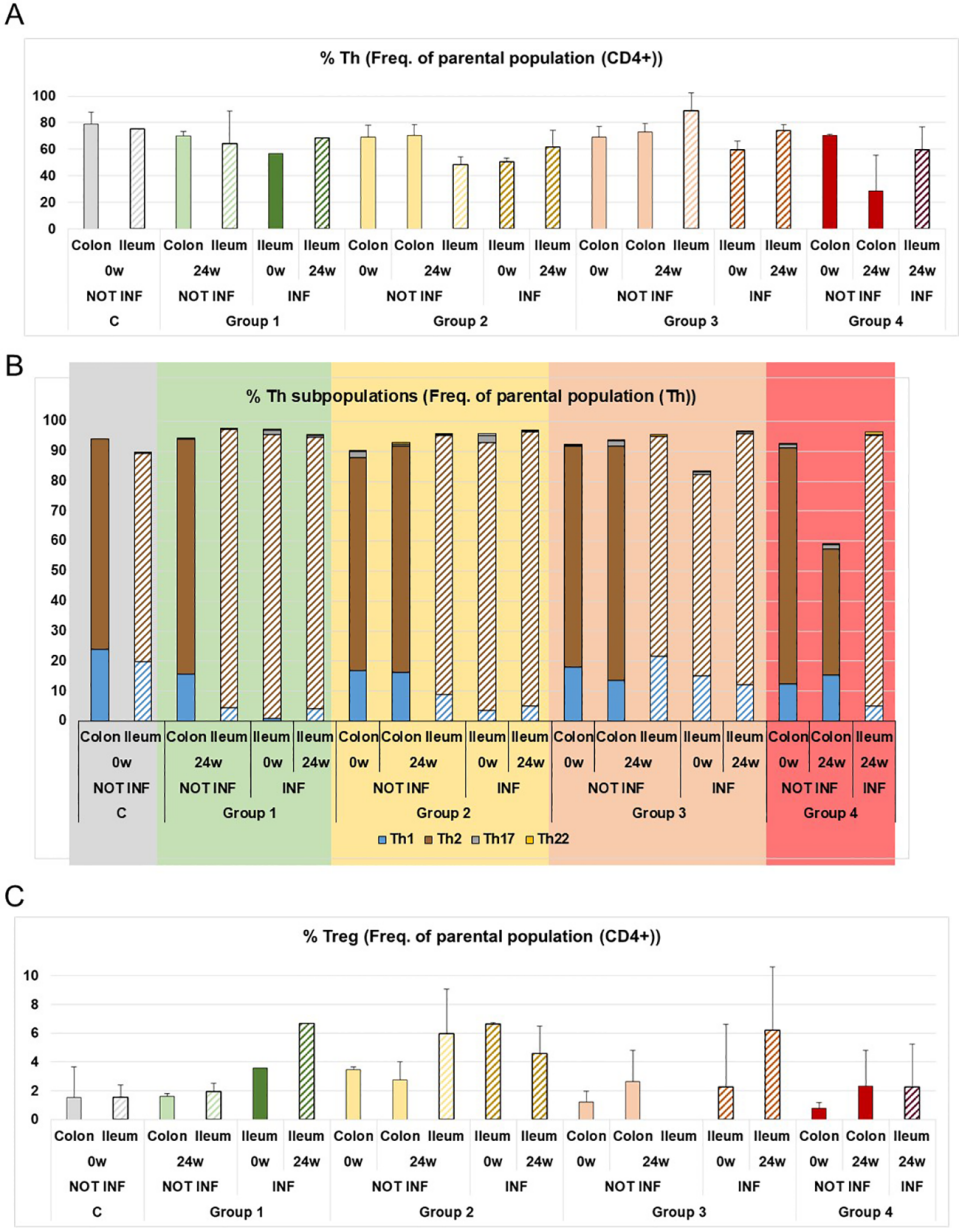
Distribution of T cells subpopulations in the lamina propria of CD patients treated with ustekinumab. **(A)** Percentage of Th cells as a percentage of the parental population, CD4+ cells. **(B)** Percentage of Th1, Th2, Th17 and Th22 cells as a percentage of the parental population, Th cells. **(C)** Percentage of Treg cells as a percentage of the parental population, CD4+ cells. C is healthy donors; 0w and 24w are 0 or 24 weeks of treatment, respectively. NOT INF, non-inflamed tissue; INF, inflamed tissue. Mean ± SD values were calculated from the measurements of all patients in each group, which in turn were obtained from the mean of three independent experiments.

Th22 cells have also been described as enhancing mucus barrier protection by promoting mucin production (**Figure 1B**) (26). Th22 levels increased in Group 1 at 24 weeks of treatment, but this result could not be confirmed as it would require testing in the lamina propria, where Th22 levels are too low to be conclusive (**Figure 2B**).

Finally, a number of studies have found that Treg cells are not altered by ustekinumab treatment (24). Observed levels did not change during the treatment in any of the Groups (**Figure 1C**). Different subpopulations of Tregs were analysed (**Supplementary Figure 2**), but their low numbers cells precluded any conclusions being drawn from these subpopulations.

From the T cell subpopulations studies in blood, we can conclude that in Group 1 (good responders), the percentages of Th subpopulations were modified during treatment, and at 24 weeks of treatment they had a similar pattern of percentages to healthy donors in terms of Th1, Th2 and Th17, while Group 4, the poor responders, showed no change to their percentages of Th subpopulations during treatment.

### Analysis of T cell subpopulations in the lamina propria of CD patients treated with ustekinumab

The next step was to analyse the T cell subpopulations in the affected tissue and measure progression over the course of treatment. To do this, we studied CD4+ T cell subpopulations in the lamina propria obtained from endoscopic biopsies in inflamed and non-inflamed tissue over a 24-week treatment (0w and 24w) (**Figure 2**). With regard to Th subpopulations, we analysed Th1, Th2, Th17 and Th22 cells (**Figures 2A, B**). This study does not permit conclusions to be drawn regarding treatment, as it is a description of Th subpopulations in the lamina propria of CD patients. Group 1 were patients with endoscopic remission and Groups 2, 3 and 4 were patients with no endoscopic remission. Regarding the colon samples, all patient samples were collected from non-inflamed tissue. We observed similar patterns of Th1 and Th2 levels between the groups and healthy donors. Despite there being non-inflamed tissues, the patients with no endoscopic remission (Groups 2, 3 and 4) harboured Th17 cells. These were not observed in Group 1 or healthy donors (**Figure 1B**). All that can be said about the ileum samples is that inflamed tissue showed Th17 levels that were not detected in non-inflamed tissues (**Figure 2B**). Finally, we detected higher levels of Treg cells in the inflamed ileum of patients with endoscopic remission (Group 1) (**Figure 2C**). Similar higher levels were detected in the inflamed ileum of Groups 2 and 3 (patients without endoscopic remission), so we cannot draw any conclusions from this observation. Different Treg subpopulations were analysed (**Supplementary Figure 3**), but the insufficient number of cells means we cannot draw any conclusions from these subpopulations.

### Analysis of cytokine levels in the serum of CD patients treated with ustekinumab

**Figure 3:**
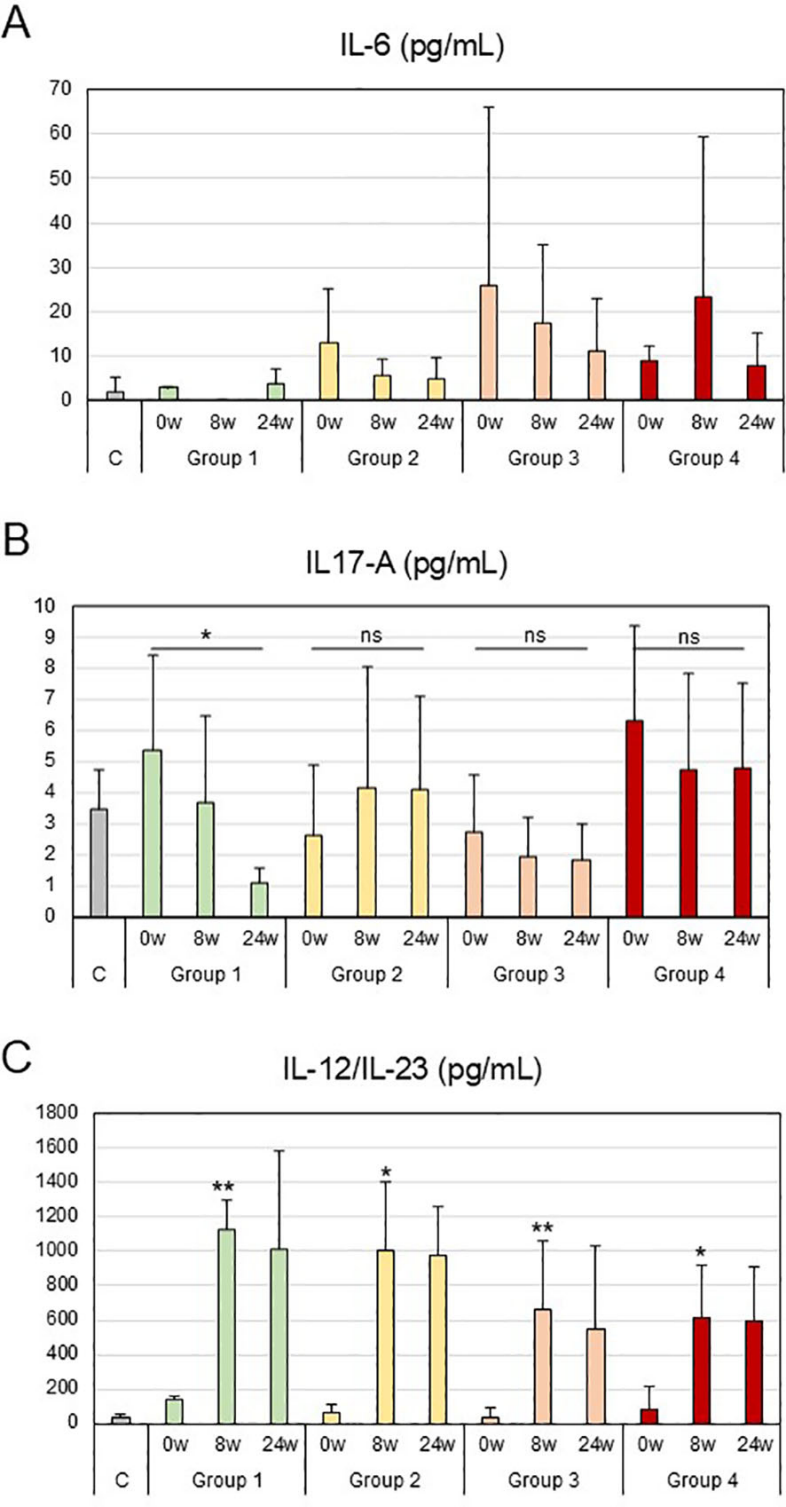
IL-6, IL-17A and IL-12/IL-23 levels in the serum of CD patients treated with ustekinumab. Production of IL-6 **(A)**, IL-17A **(B)** and IL-12/IL-23 **(C)**, as assessed by ELISA, in the serum from patients (Groups 1-4) and healthy controls **(C)** at 0, 8 and 24 weeks of treatment. Mean ± SD values were calculated from the measurements of all patients in each group, which in turn were obtained from the mean of three independent experiments. Friedman’s test for dependent samples was used for the studies of IL-17-A, comparing the differences between week 0 and week 24 of treatment for each group; *p* = 0.05 (*) for the difference between week 0 and week 24 of Group 1; and non-significant (ns) for the difference between week 0 and week 24 of Groups 2, 3 and 4. Tukey’s multiple comparisons test was used for the studies of IL-12/IL-23 comparing the differences between healthy controls and the patient groups at 8 weeks of treatment; *p* = 0.002 (**) for the difference between Group 1 and C; *p* = 0.02 (*) for the difference between Group 2 and C; *p* = 0.005 (**) for the difference between Group 3 and C; and *p* = 0.03 (*) for the difference between Group 4 and C.

The last step was to analyse the levels of different cytokines in the patient serum to monitor their progression during treatment with ustekinumab. We measured IL-6, IL-17 and IL-12/IL-23 in patient serum over a 24-week treatment period (0w, 8w and 24w) (**Figure 3**). With regard to IL-6 levels, Group 1 had the lowest levels, being the patients with the best response to treatment (**Figure 3A**). For IL-17 levels, Group 1 showed a steady, statistically significant drop in levels of this cytokine during treatment, while the groups with a poorer response showed no significant reduction in IL-17 levels (**Figure 3B**). IL-12/IL-23 was the cytokine blocked by ustekinumab, as shown in **Figures 3C**, where levels of IL-12/IL-23 rose after the treatment in all groups. Group 1, with the best response to treatment, maintained the highest levels of IL-12/IL-23 in serum during treatment, thus proving it is the group in which the treatment was most efficient, with the highest statistically significant differences compared to healthy donors at 8 weeks of treatment, generating the highest block of IL-12/IL-23 receptors. By contrast, Group 4 had the lowest levels of IL-12/IL-23 in serum, showing that blocking is not as efficient in these patients. There was a correlation between IL-12/IL-23 levels in serum and the response to treatment: the higher the levels of IL-12/IL-23 after treatment, the better the response to ustekinumab treatment.

## Discussion

CD affects from 6 to 10 million people worldwide (27, 28). In recent years, treatment of IBD has been revolutionised by the introduction of therapeutic antibodies, such as anti-IL-12/IL-23 antibodies (19). These antibodies have been developed to block the IL-12/IL-23 signalling pathway in order to control the immune response (19). In this study, we aimed to fully characterise different immune parameters, such as T cell subpopulations and cytokine expression, in CD patients with different response profiles to treatment with anti-IL-12/IL-23. This would then help to identify predictors for early evaluation of whether the treatment is going to be effective, switching to the next line of treatment if not. By doing so, the time required to find the optimal treatment of patients could be reduced, thereby providing them with personalised medicine. Regarding the T cell subpopulation study, an exhaustive cytometric analysis of cellular subpopulations was carried out both in peripheral blood and in the lamina propria throughout the progression and treatment of the disease, distinguishing between the different responses to treatment. The main limitations of this study include the relatively small sample size, the imbalance between response groups, and the exploratory nature of the analyses, which may limit the robustness and generalizability of the conclusions. The peripheral blood analysis showed that Th1 levels drop and Th2 levels rise throughout the course of treatment in patients with a good response to IL12/IL23 inhibitor treatment, contrary to poor-responder patients. In addition, we observed high Th17 levels in poor responders that did not drop after treatment (**Table 3**). The studies of T cell subpopulations in peripheral blood are not conclusive, but they could guide clinicians with regard to the patient’s response to treatment (**Table 3**). Larger numbers of patients are required to draw firmer conclusions.

**Table 3:** Summary of the key points that can be used as predictors of a good response to anti-IL12/IL-23 treatment.

Good responders to anti-IL-12/IL-23	
Sample and time of treatment	Key points
PBMC – 24 weeks	Reduction of Th1 levels
PBMC – 24 weeks	Increased of Th2 levels
Serum – 24 weeks	Reduction of IL-17 levels
Serum – 8 weeks	High levels of IL-12/IL-23

In terms of cytokine levels, production of IL-6 was observed to provide an initial indication of the patient’s clinical status. Although these data are difficult to extrapolate, given the low concentration of IL-6, it was observed that the lowest levels occurred in good responders. A good biomarker is the level of IL-17, as good responders to treatment had significantly lower levels of this cytokine at 24 weeks of treatment, a pattern that did not appear in the other groups (**Table 3**). The earliest biomarker was serum IL-12/IL-23 levels: good responders had higher levels of this cytokine at 8 weeks of treatment, due to effective blocking of anti-IL-12/IL-23. A correlation was observed in which the worse the responders, the lower the serum levels of IL-12/IL-23. This may be because the receptors are not well blocked, thereby indicating that the treatment will not be effective. However, it could also be showing that the IL-12/IL-23 signalling pathway is not the only or even the main one affected, suggesting that other pathways should be blocked for effective treatment.

Based on all the above results, this study provides evidence that immune signatures may help predict response to IL-12/IL-23 blockade in Crohn’s disease. In peripheral blood, good responders displayed reduced Th1, increased Th2, and lower IL-17A levels by week 24, whereas poor responders do not have these profiles. Importantly, higher serum IL-12/IL-23 levels as early as week 8 correlated with favourable outcomes, reflecting effective pathway inhibition (**Table 3**). These findings suggest that monitoring serum Th1, Th2 and IL-12/IL-23 may represent promising candidate biomarkers for early assessment of ustekinumab response in CD, which require validation in larger prospective cohorts. Overall, this study should be considered exploratory and hypothesis-generating. Our results provide a rationale for future mechanistic and validation studies in larger, multicentre cohorts before these immune signatures can be considered for clinical use being an advance in precision medicine in inflammatory bowel disease.

## Data Availability

The original contributions presented in the study are included in the article/**Supplementary Material**. Further inquiries can be directed to the corresponding authors.
