# Supplementary material for: T cell and cytokine signatures as early predictors of response to IL-12/IL-23 inhibition in Crohn’s disease
Source: Front Immunol. 2026 Mar 4;17:1753914. doi: 10.3389/fimmu.2026.1753914 (PMC12996213; doi:10.3389/fimmu.2026.1753914)
Supplement: Supplementary file 1 [file SupplementaryFile1.docx]

**This PDF file includes**

**Figures S1 to S3**

**Tables S1 and S3**

**FIGURES**

**Figure S1. Flow cytometry gating strategy.**

**
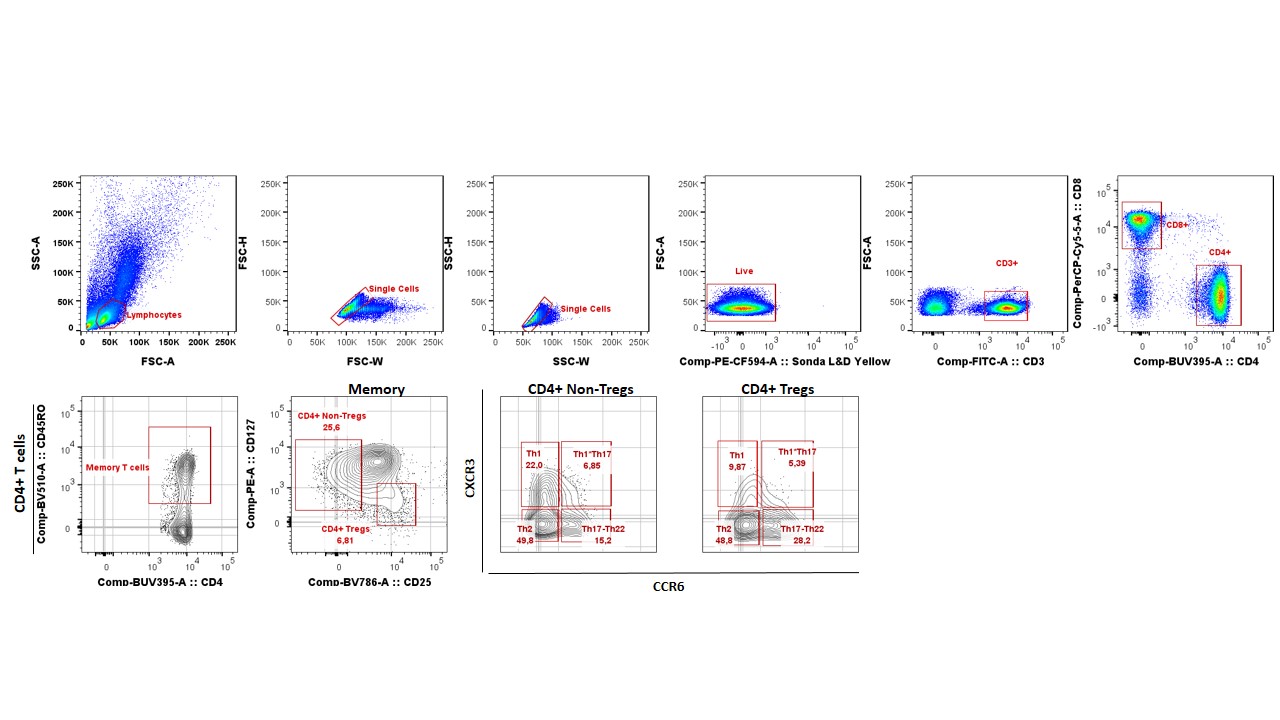
**

**Figure S2. Distribution of Treg subpopulations in the PBMC of CD patients treated with ustekinumab**

**
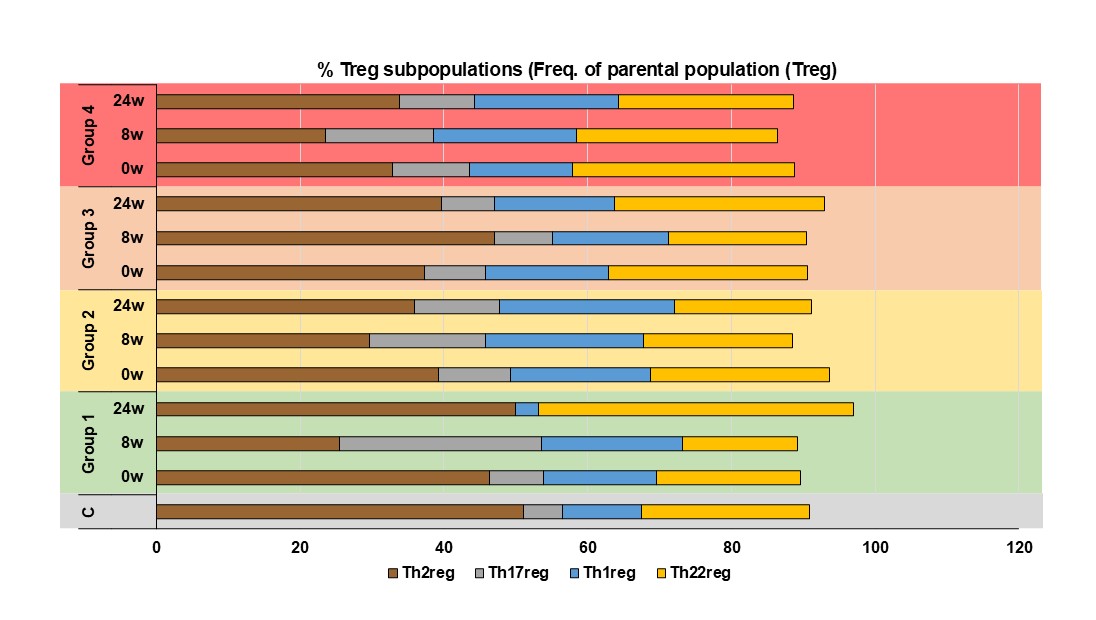
**

**Figure S2. Distribution of Treg subpopulations in the PBMC of CD patients treated with ustekinumab**

Percentage of Th1reg, Th2reg, Th17reg and Th22reg cells as a percentage of the parental population, Treg cells. C is healthy donors, 0w, 8w and 24w are 0, 8 or 24 weeks of treatment, respectively. Mean ± SD values were calculated from the measurements of all patients in each group, which in turn were obtained from the mean of three independent experiments.

**Figure S3. Distribution of Treg cells subpopulations in the lamina propria of CD patients treated with ustekinumab**

**
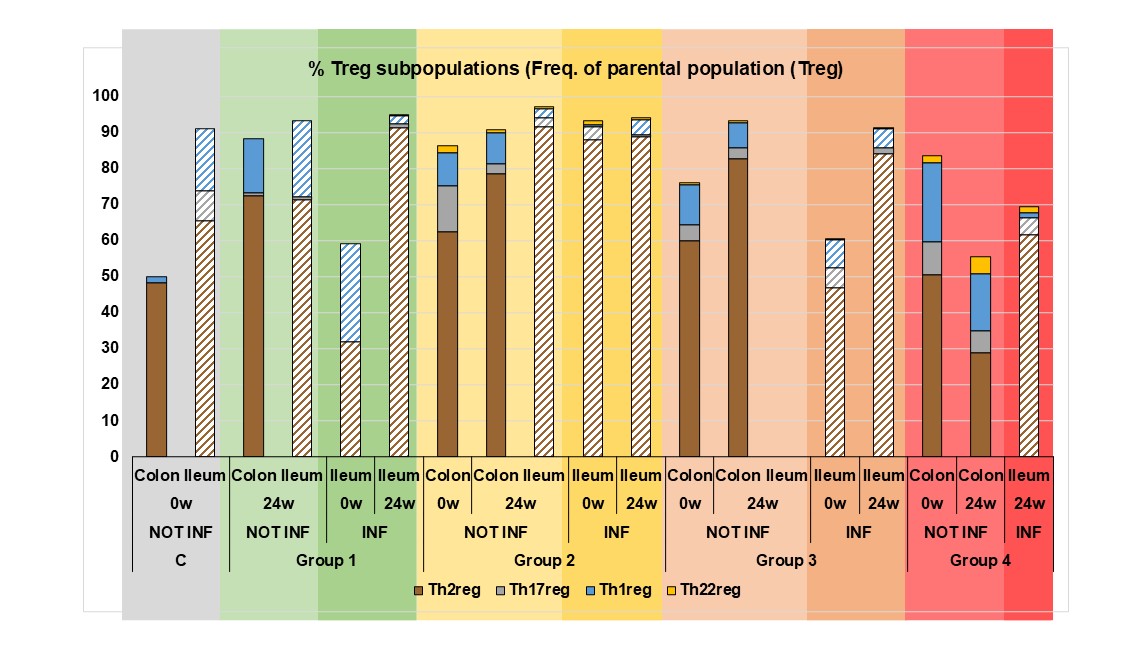
**

**Figure S3. Distribution of Treg cells subpopulations in the lamina propria of CD patients treated with ustekinumab**

Percentage of Th1reg, Th2reg, Th17reg and Th22reg cells as a percentage of the parental population, Treg cells. C is healthy donors, 0w and 24w are 0 or 24 weeks of treatment, respectively. NOT INF: non-inflamed tissue; INF: inflamed tissue. Mean values ± SD were calculated from the measurements of all patients in each group, which in turn were obtained from the mean of three independent experiments.

**TABLES**

**Table S1. Analytical data of patients include in the study**

**Table S1. Analytical data of patients include in the study.** Levels of the inflammatory markers calprotectin, C-reactive protein and albumin in patients tested prior to treatment (WEEK 0), at 8 weeks of treatment (WEEK 8) and 24 weeks of treatment (WEEK 24). The Harvey-Bradshaw Index (HBI)^1^ and Simple Endoscopic Score for Crohn’s Disease (SES-CD)^2^ used to stratify severity of Crohn’s disease are also included. Drug levels show the free fraction of ustekinumab measured in peripheral blood (antibody-unbound) at 8 and 24 weeks of treatment.

**Table S2. Response to treatment**

**Table S2. Response to treatment.** Response to treatment measured as clinical, biological and endoscopic remission in the CD patients included in the study at 8 and 24 weeks of treatment.

**Table S3. Demographic and clinical characteristics (mean)**

| **Variable** | N = 23 |
| --- | --- |
| **Age** | 52.8 (16 - 82) |
| **Sex** |  |
| Female | 14 (60.9%) |
| Male | 9 (39.1%) |
| **CD behaviour according to Montreal** |  |
| B1 | 7 (30.4%) |
| B2 | 16 (69.6%) |
| B3 | 0 (0%) |
| **CD location according to Montreal** |  |
| L1 | 11 (47.8%) |
| L2 | 0 (0%) |
| L3 | 12 (52.2%) |
| **Perianal disease** |  |
| No | 19 (82.6%) |
| Yes | 4 (17.4%) |
| **Upper involvement** |  |
| 0 | 21 (91.3%) |
| 1 | 2 (8.7%) |
| **Extraintestinal manifestations** |  |
| No | 19 (82.6%) |
| Yes | 4 (17.4%) |
| **Concomitant Medication** |  |
| No | 19 (82.6%) |
| Corticosteroid | 0 (0%) |
| Immunosuppressant | 4 (17.4%) |
| **Smoking** |  |
| No | 14 (60.9%) |
| Yes | 9 (39.1%) |
| **CVRF** |  |
| No | 16 (69.6%) |
| Yes | 7 (30.4%) |
| **Weight (Kg)** | 69.2 (43.1 – 103.5) |
| **HBI** | 2.6 (0 - 13) |
| **IBDQ-9** | 42 (27 – 63) |

**REFERENCES:**

1. Harvey RF., Bradshaw MJ. Measuring Crohn’s disease activity. *Lancet (London, England)* 1980:1134–5. Doi: 10.1016/s0140-6736(80)91577-9.

2. Daperno M., D’Haens G., Van Assche G., Baert F., Bulois P., Maunoury V., et al. Development and validation of a new, simplified endoscopic activity score for Crohn’s disease: the SES-CD. *Gastrointest Endosc* 2004;**60**(4):505–12. Doi: 10.1016/s0016-5107(04)01878-4.
